# Role of *UCP1* Gene Variants in Interethnic Differences in the Development of Cardio-Metabolic Diseases

**DOI:** 10.3389/fgene.2017.00007

**Published:** 2017-01-30

**Authors:** Andreas D. Flouris, Yulii V. Shidlovskii, Alexander V. Shaposhnikov, Levon Yepiskoposyan, Liliya Nadolnik, Lidia Karabon, Anna Kowalska, Andres E. Carrillo, George S. Metsios, Paraskevi Sakellariou

**Affiliations:** ^1^FAME Laboratory, Institute of Research and Technology Thessaly, Centre for Research and Technology HellasTrikala, Greece; ^2^Institute of Gene Biology – Russian Academy of SciencesMoscow, Russia; ^3^National Academy of Sciences of the Republic of ArmeniaYerevan, Armenia; ^4^Institute of Biochemistry of Biologically Active Compounds – National Academy of Sciences of BelarusGrodno, Belarus; ^5^Institute of Immunology and Experimental Therapy – Polish Academy of SciencesWrocław, Poland; ^6^Department of Exercise Science, Chatham University, PittsburghPA, USA; ^7^Faculty of Education, Health and Wellbeing, Wolverhampton UniversityWalsall, UK

**Keywords:** gene variants, cardio-metabolic diseases, uncoupling protein 1, brown adipose tissue, metabolism, prevalence rates, ethnic groups, population studies

## Abstract

Cardio-metabolic diseases (CMDs) comprise a cluster of risk factors that contribute to chronic pathological conditions with adverse consequences for cardiovascular function and metabolic processes. A wide range of CMD prevalence rates among different ethnic groups has been documented. In view of accumulated evidence, there is a trend toward increasing CMD prevalence rates in Eastern Europe and Western Asia. Numerous studies have revealed an association between uncoupling protein 1 (*UCP1*) gene variants and CMDs. UCP1 activity is essential for brown adipose tissue (BAT)-mediated thermogenesis. Experimental animal studies and epidemiological studies in humans highlight the significance of BAT-mediated thermogenesis in protecting against obesity and maintaining a lean phenotype. We hypothesize that the genetic variation in *UCP1* gene expression observed among different ethnic groups could contribute to the ethnic-specific predisposition to CMD development. Constructing such prevalence maps of *UCP1* gene variants could contribute significantly into identifying high-risk ethnic groups predisposed to the development of CMDs, and further shaping public health policies by the improvement of existing preventive and management strategies.

## Introduction

**FIGURE 1:**
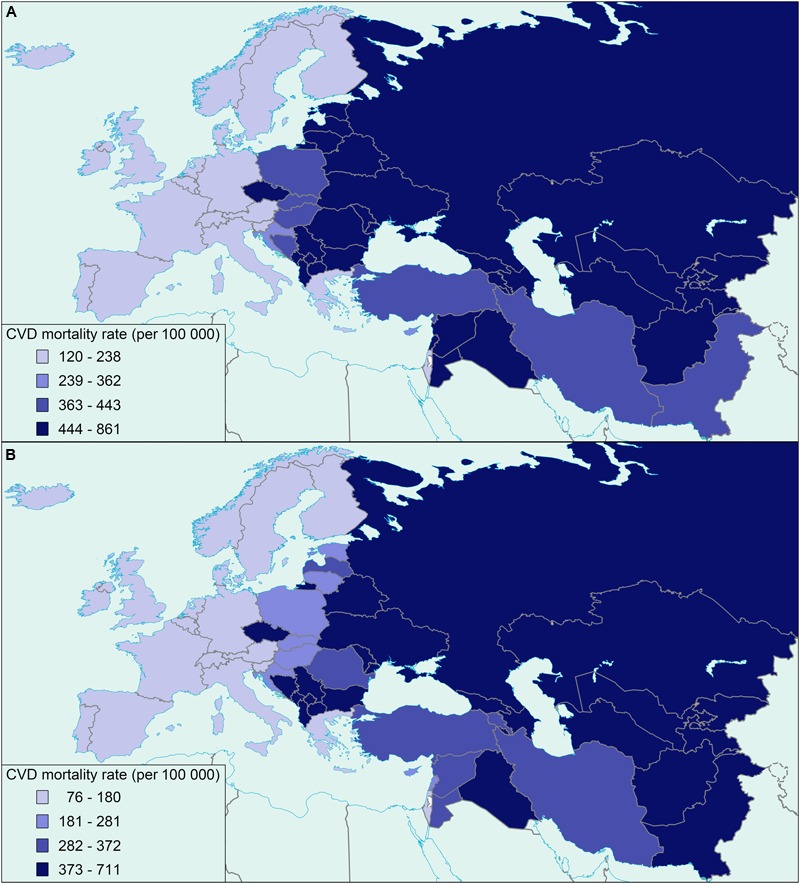
**Map of Europe and Western Asia showing the distribution of cardiovascular mortality rates in males **(A)** and females (B)**, adapted from the World Health Organization (WHO, 2011) (age standardized, per 100 000).

The World Health Organization (WHO) estimates that by 2030 more than 23 million patients affected by cardiovascular diseases (CVDs) – the leading cause of death globally – will die annually (WHO, 2016a), while diabetes will become the seventh leading cause of death (WHO, 2016b). In total, the European region has one of the highest mortality rates for adults under the age of 70 years due to a very high prevalence of cardio-metabolic diseases (CMDs) (Mathers et al., 2009; WHO, 2014). CMDs comprise a cluster of risk factors that increase susceptibility to developing CVDs, such as atherosclerosis and arterial thrombosis, metabolic syndrome, and type 2 diabetes mellitus, and its related metabolic traits. CMD-associated risk factors among others include obesity, hypertension, low high-density lipoprotein cholesterol, insulin resistance, glucose intolerance, and elevated triglycerides (Grundy et al., 2004; Cornelissen and Smart, 2013; Vissers et al., 2013; Conn et al., 2014). Recently, the prevalence of obese individuals with metabolic syndrome in a large cohort of participants across Europe was estimated to reach as high as 78 and 65% in males and females, respectively (van Vliet-Ostaptchouk et al., 2014). Another example is a trend toward the increase in CVD prevalence in certain ethnic groups in Eastern Europe and Western Asia compared to Western Europe (**Figure 1**) (Balkau et al., 2007; WHO, 2011).

The uncoupling protein-1 (*UCP1*) gene is located in chromosome 4 of the human genome and is considered to be involved in the pathogenesis of CMDs due to its major role in thermogenesis and energy metabolism (Golozoubova et al., 2001; Mattson, 2010). UCP1 is mainly expressed in brown adipose tissue (BAT) and is located in the inner mitochondrial membrane where it catalyzes proton leaks across the membrane, and thus uncoupling oxidative phosphorylation from ATP production (Tsuboyama-Kasaoka et al., 1998). This is proposed to lead to energy dissipation in the form of heat, accompanied by an increase in energy expenditure (Wu et al., 2013). It has been suggested that BAT activity, mediated by UCP1 expression, in humans can contribute to 5% of the basal metabolic rate (van Marken Lichtenbelt and Schrauwen, 2011), indicative of a regulatory role of BAT in energy balance and hence body weight. In adult humans, the levels of UCP1 in BAT are diminished with age and are negatively correlated to adiposity (Saito et al., 2009; van Marken Lichtenbelt et al., 2009). Indeed, individuals with low levels of BAT activity are more susceptible to developing CMDs (Mattson, 2010) and, as we previously proposed, the rate of aging is determined, at least in part, through changes in BAT activity (Carrillo and Flouris, 2011; Flouris and Piantoni, 2015). In addition to age- and adiposity-associated loss of BAT activity, *UCP1* gene variants may also increase one’s tendency for developing CMDs by disrupting regular BAT function (Jia et al., 2010). In this light, BAT activation is impaired in healthy individuals carrying specific *UCP1* gene variants (Nagai et al., 2007), corroborating a role of *UCP1* gene variation in adverse metabolic processes and susceptibility to CMDs. Experimental animal evidence supports a relationship between BAT activity and energy homeostasis. For instance, inactivation of *UCP1* due to thermoneutral conditions led to obesity in mice fed both a control diet and high-fat diet, indicating a role of BAT in maintaining a non-obese phenotype (Feldmann et al., 2009). On the other hand, overexpression of *UCP1* in white adipose tissue and skeletal muscle of mice, provided protection against diet-induced obesity, increased energy expenditure, and improved glucose homeostasis (Keipert et al., 2013; Ost et al., 2014). In addition, embryonic BAT transplants have been shown to reverse type 1 diabetes in mice in an insulin receptor activity-dependent mechanism suggesting an important role of BAT in whole-body metabolism (Gunawardana and Piston, 2012).

**FIGURE 2:**
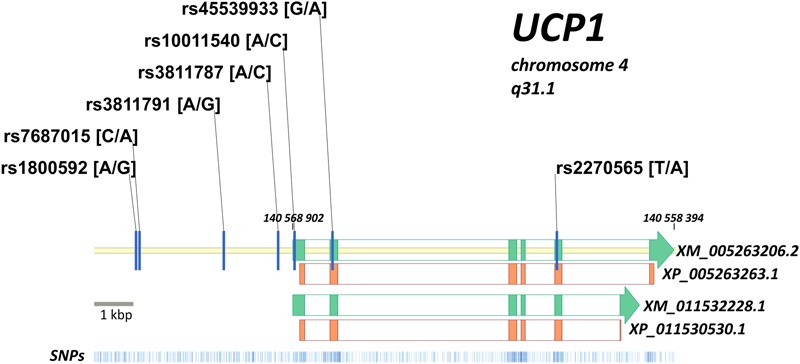
**Intron–exon structure of the *UCP1* gene is shown (green blocks) according to two predicted transcripts (XM_005263206.2 and XM_011532228.1 with corresponding protein forms XP_005263263.1 and XP_011530530.1).** Protein coding regions are shown by orange blocks, coding sequence of *UCP1* gene is in reverse chromosome DNA strand. Position of XM_005263206.2 in genome is indicated (140,559,394 … 140,568,902 bp). Seven mostly investigated variants of the UCP1 gene are located on the map: -3826A/G (rs1800592), -3737C/A (rs7687015), -1766A/G (rs3811791), -412A/C (rs3811787), -112A/C (rs10011540), Arg64Thr (rs45539933), Met229Leu (rs2270565). Distribution of known genetic variants along the locus is shown at the bottom (blue bars).

The current NCBI database of genetic variations shows more than 2300 single nucleotide polymorphisms (SNPs) associated with the *UCP1* gene (Sherry et al., 2001). The intron–exon structure of the *UCP1* gene is illustrated in **Figure 2**. Several studies have investigated the prevalence of such *UCP1* gene variants and their influence on the susceptibility to CMDs, though data still remain scarce among different ethnic groups in Europe. Indeed frequencies for most *UCP1* gene variants have been estimated in selective ethnic groups with small number of participants, rarely reaching more than a few hundreds. Further, the available studies have focused on certain polymorphisms -3826A/G, -112A/C, and -1766A/G in the 5′-region, Ala64Thr in exon 2, and Met229Leu in exon 5 of the gene (reviewed in Jia et al., 2010; Brondani et al., 2012). In addition, emerging evidence of CVD prevalence rates being the highest globally among ethnic groups in Eastern Europe and Western Asia (Balkau et al., 2007), suggests investigation of the effect of *UCP1* gene variants on CMD predisposition across these regions. Therefore, accurate estimation of the prevalence of the most commonly studied *UCP1* gene variants and identification of ethnic groups at high risk of developing CMDs warrants further analysis in large cohorts of patients and healthy controls. Integration of the findings to national public health policies may substantially improve CMD preventive and management strategies.

## The Hypothesis of the *UCP1* Genetic Variation Effect on the Interethnic Differences in CMD Prevalence Rates

On the grounds of experimental evidence corroborating an association of *UCP1* gene variants and CMDs, we hypothesize that the ethnic-specific predisposition to CMDs could be attributed, at least partially, to the diverse prevalence of specific *UCP1* gene variants among different ethnic groups. Such an increased susceptibility to the development of CMDs has been observed specifically in ethnic groups in Eastern Europe and Western Asia (**Figure 1**). Large population-based studies are thus required to investigate the CMD-related association of specific *UCP1* gene variants among different ethnic groups. Information on ethnic-specific genotypes’ frequencies of the analyzed *UCP1* gene variants could be used to identify those ethnic groups at high-risk of developing CMDs, and further explain to an extent the high CMD prevalence rates observed in Eastern Europe and Western Asia.

### Evaluation of the Ethnic-Specific *UCP1* Gene Variants in CMD Susceptibility and Prevalence Rates

Due to its aforementioned role in the regulation of energy metabolism, UCP1 has been considered a potential genetic risk factor for predisposition to CMDs (Feldmann et al., 2009; Jia et al., 2010). For that reason, numerous genetic association studies in selective European populations have been performed to explore a potential link between *UCP1* gene variants and CMDs, often yielding to controversial results. A number of independent genetic association studies suggested an association between *UCP1* gene variants and obesity, type 2 diabetes mellitus, body fat distribution, and metabolic syndrome-related traits (Clement et al., 1996; Hamann et al., 1998; Kiec-Wilk et al., 2002; Forga et al., 2003; Herrmann et al., 2003; Ramis et al., 2004; Sramkova et al., 2007), while other studies failed to demonstrate such correlations (Urhammer et al., 1997; Fogelholm et al., 1998; Gagnon et al., 1998; Sivenius et al., 2000; Nieters et al., 2002; Malczewska-Malec et al., 2004; Mottagui-Tabar et al., 2008). The most commonly studied *UCP1* gene variant is -3826A/G, while the synergistic effect of several variants in *UCP1* gene has been investigated to a lesser extent (reviewed in Brondani et al., 2012). To our knowledge, there has been no genetic association study to investigate the combined effect of the most common *UCP1* gene variants on the susceptibility to CMDs among different ethnic groups in Europe.

On the other hand, differences in the number of UPC1 polymorphism carriers have been reported in various populations (Jia et al., 2010). However, given the relatively low prevalence of UCP1 gene polymorphisms [can be as low as 2 or 6% for specific polymorphisms (Vimaleswaran et al., 2007)], these data are limited by the small sample sizes assessed (<500 participants) (Kogure et al., 1998; Hayakawa et al., 1999; Nakano et al., 2006). Small study populations limit the accuracy of genotype prevalence estimates (e.g., only 10 identified individuals in a study of 200 participants with a 5% UCP1 gene polymorphism prevalence rate), making it difficult to integrate the evidence and, thus, to easily translate the findings into public health improvements (Ioannidis et al., 2001; Chang et al., 2009). More recent studies investigating the prevalence of genetic polymorphisms and their impact on health and disease included thousands of participants among diverse populations (Yoshida et al., 2009). Given also the increased CMD prevalence rates specifically in ethnic groups in Eastern Europe and Western Asia, it is expected that large case-control, population-based studies will identify those *UCP1* gene variants that predispose ethnic groups across these regions to CMD development. Despite their increased cost, such studies will potentially explain the highest CMD prevalence rates observed across these regions at a global level.

Further studies to investigate a synergistic effect of the studied *UCP1* gene variants with environmental (geographical location, climate, diet, and physical activity) and/or socio-economic (education, income, and cultural conception) factors could significantly contribute to define the association between the genetic variation in *UCP1* gene and CMDs, by fully adjusting for such potential confounders. The emerging field of human social genomics highlights the importance of the contemporary dynamic social adverse conditions on influencing ones genotype and hence further shaping the susceptibility to chronic diseases. On the other hand, gene variants can also alter genotypic response to socio-environmental adversities by activating different molecular pathways and factors, and thus enhancing or reducing the risk for chronic disease (Slavich and Cole, 2013). Future investigation to address such dynamic effects of external social environment on *UCP1* gene expression may also reveal additional factors, such as experience of chronic social isolation and isolation of obese individuals, which could in turn either enhance or reduce the impact of specific *UCP1* gene variants on ethnic-specific predisposition to CMDs.

We propose to investigate the putative association of the several *UCP1* gene variants with the development of CMDs. All patients and volunteers in this study will derive from six different ethnic groups, with ethnicity to be defined by self-report. Our sample population will be comprised of 1500 unrelated individuals aged of >18 years diagnosed with type-2 diabetes, obesity, and metabolic syndrome with or without hypertension. Our control group will contain 1500 age- and gender-matched healthy individuals. Exclusion criteria will include individuals < 18 years of age, smoking, history of eating disorder, pregnancy or lactation, acute illness and/or infection (last 4 weeks). Participants will be white Caucasians of European and Western Asian origin. They will be weighed, their height will be measured, and their BMI will be calculated. Blood pressure will be assessed. Percent body fat will be measured three times, and waist-to-hip ratio will be calculated. Further, after an overnight fast, plasma glucose will be measured. Environmental and socio-economic factors will be determined by using standardized questionnaires. The seven most commonly studied *UCP1* gene variants (**Figure 2**) will be genotyped. Genomic DNA will be extracted from whole blood and each *UCP1* gene variant will be assessed by Real Time Taqman method. To our knowledge only three studies have conducted *in vitro* or *in vivo* studies to characterize the function of two specific *UCP1* gene variants (rs1800592 and rs7687015) (Esterbauer et al., 1998; Rose et al., 2011; Brondani et al., 2012). We aim, therefore, to assess the putative functional relevance of specific alleles and haplotypes of the aforementioned variants on the transcriptional activity of *UCP1* by *in vitro* reporter assays. Appropriate statistical analysis will be used to investigate the associations of *UCP1* variant-specific alleles and haplotypes with CMDs, and in relation with clinical characteristics. The modifying effect of environmental factors, with a special focus on diet variation among the different ethnic groups studied, on the association between CMDs and UCP1 gene variants will also be examined. Further, the effect of socio-economic factors on *UCP1* transcriptional activity as putatively mediated by the analyzed alleles and haplotypes of *UCP1* gene variants will also be determined.

This proposed investigation of the combined effect of the most commonly studied *UCP1* gene variants on CMD prevalence in a very large and ethnically diverse sample of patients and healthy individuals will identify high-risk ethnic groups for developing CMDs. Further, the study of *UCP1* gene variants-environment interactions will define a strong association between *UCP1* gene variants and CMD development.

## Assessment of the Clinical Implications and the Impact of *UCP1* Gene Variants on Public Health Care System, Policy and Preventive Practices

Cardio-metabolic diseases constitute a serious public health concern with a substantial impact not only on life expectancy (Calle and Kaaks, 2004; Poirier et al., 2006; WHO, 2011, 2014; Cornelissen and Smart, 2013; Conn et al., 2014) but also on healthcare services at a major cost to the economy (WHO, 2007). A substantial economic pressure on healthcare systems is imposed by CVDs across Europe, reaching €196 billion per year (Nichols et al., 2012). Recent reports predict a 33% increase in the global market for drugs treating CVDs from 2012 to 2019 (Gbi research, 2013). Further, USA comprise the biggest single market for anti-obesity drugs, with around 68% of the population either overweight or obese, followed by the UK and other European countries (Holvoet, 2012).

Despite the growing cost of anti-CMD agents, there is concern that the drugs fail to provide lasting benefits for health and well-being (Huntington and Shewmake, 2010). In this light, identification of interethnic differences in *UCP1* gene variants that could predispose certain ethnic groups to the development of CMDs could assist in the design of effective preventive and management practices, with beneficial outcomes for the public health care services. *UCP1* gene variants have been associated with high levels of low-density lipoprotein (LDL) cholesterol and diastolic blood pressure, as well as with low levels of high-density lipoprotein (HDL) cholesterol (Kiec-Wilk et al., 2002; Oh et al., 2004). In turn, high levels of circulating LDL cholesterol are implicated in the development of atherosclerosis and represent important pharmacological target in preventing the progression of atherosclerosis and event of coronary heart disease and stroke (Takahashi et al., 2010). Therefore, it is evident that a fresh perspective in targeted interventions is necessary to efficiently manage the massive projected increase of CMDs over the next decades.

The diverse distribution of CMDs among different ethnic groups could be also attributed to environmental factors, and socio-economic factors. WHO estimates that more than 80% of CVDs and diabetes deaths occur in low-and middle-income countries (WHO, 2016a,b). It is therefore crucial to determine the potential modifying effects of the environmental factors on the *UCP1* gene variation risk pattern of CMD development. Lastly, in light of a growing body of evidence, the once accepted conception on an otherwise stable and impermeable genetic make-up has been challenged, supporting reciprocal gene-adverse social environment interactions, with putative strong impact on the role of certain UCP1 gene variants in defining ethnic-specific susceptibility to CMDs.

## Conclusion

The association between specific *UCP1* gene variants and CMDs has been investigated in certain ethnic groups, often leading to controversial results. There is the need to improve our understanding on the prevalence of the most commonly studied *UCP1* gene variants among various ethnic groups and their impact on the predisposition to CMDs. It can be hypothesized that interethnic differences in the distribution of *UCP1* gene variants could account, at least in part, for such a diverse population-specific predisposition to CMDs. One such example of a wide variation in CMD prevalence rates has been observed across Europe and Western Asia. Addressing this hypothesis in multi-ethnic based studies will provide information on the functional effect of these genetic variants on the pathogenetic mechanisms of CMDs. Identifying high-risk ethnic groups of developing CMDs will set the ground for defining public health policies and improving existing preventive and management strategies.

## Author Contributions

AF (corresponding author) was responsible for the conception and design of the work and was involved in drafting and revising the manuscript. PS was responsible in drafting and revising the manuscript. LY, LN, LK, AK, AS, AC, GM, and YS participated in the conception of the work, read and approved the final manuscript.

## Conflict of Interest Statement

The authors declare that the research was conducted in the absence of any commercial or financial relationships that could be construed as a potential conflict of interest.

## References

[B1] Balkau B. Deanfield J. E. Despres J. P. Bassand J. P. Fox K. A. Smith S. C. (2007). International day for the evaluation of abdominal obesity (idea): a study of waist circumference, cardiovascular disease, and diabetes mellitus in 168,000 primary care patients in 63 countries. *Circulation* 116 1942–1951. 10.1161/circulationaha.106.676379 17965405 PMC2475527

[B2] Brondani L. A. Assmann T. S. Duarte G. C. Gross J. L. Canani L. H. Crispim D. (2012). The role of the uncoupling protein 1 (UCP1) on the development of obesity and type 2 diabetes mellitus. *Arq. Bras. Endocrinol. Metabol.* 56 215–225. 10.1590/S0004-27302012000400001 22790465

[B3] Calle E. E. Kaaks R. (2004). Overweight, obesity and cancer: epidemiological evidence and proposed mechanisms. *Nat. Rev. Cancer* 4 579–591. 10.1038/nrc1408 15286738

[B4] Carrillo A. E. Flouris A. D. (2011). Caloric restriction and longevity: effects of reduced body temperature. *Ageing Res. Rev.* 10 153–162. 10.1016/j.arr.2010.10.001 20969980

[B5] Chang M. H. Lindegren M. L. Butler M. A. Chanock S. J. Dowling N. F. Gallagher M. (2009). Prevalence in the united states of selected candidate gene variants: third national health and nutrition examination survey, 1991-1994. *Am. J. Epidemiol.* 169 54–66. 10.1093/aje/kwn286 18936436 PMC2638878

[B6] Clement K. Ruiz J. Cassard-Doulcier A. M. Bouillaud F. Ricquier D. Basdevant A. (1996). Additive effect of A– > G (-3826) variant of the uncoupling protein gene and the Trp64Arg mutation of the beta 3-adrenergic receptor gene on weight gain in morbid obesity. *Int. J. Obes. Relat. Metab. Disord.* 20 1062–1066. 8968850

[B7] Conn V. S. Koopman R. J. Ruppar T. M. Phillips L. J. Mehr D. R. Hafdahl A. R. (2014). Insulin sensitivity following exercise interventions: systematic review and meta-analysis of outcomes among healthy adults. *J. Prim. Care Community Health* 5 211–222. 10.1177/2150131913520328 24474665 PMC4393364

[B8] Cornelissen V. A. Smart N. A. (2013). Exercise training for blood pressure: a systematic review and meta-analysis. *J. Am. Heart Assoc.* 2:e004473 10.1161/jaha.112.004473 PMC3603230 23525435

[B9] Esterbauer H. Oberkofler H. Liu Y. M. Breban D. Hell E. Krempler F. (1998). Uncoupling protein-1 mRNA expression in obese human subjects: the role of sequence variations at the uncoupling protein-1 gene locus. *J. Lipid Res.* 39 834–844. 9555947

[B10] Feldmann H. M. Golozoubova V. Cannon B. Nedergaard J. (2009). UCP1 ablation induces obesity and abolishes diet-induced thermogenesis in mice exempt from thermal stress by living at thermoneutrality. *Cell Metab.* 9 203–209. 10.1016/j.cmet.2008.12.014 19187776

[B11] Flouris A. D. Piantoni C. (2015). Links between thermoregulation and aging in endotherms and ectotherms. *Temperature* 2 73–85. 10.4161/23328940.2014.989793 PMC4843886 27226994

[B12] Fogelholm M. Valve R. Kukkonen-Harjula K. Nenonen A. Hakkarainen V. Laakso M. (1998). Additive effects of the mutations in the beta3-adrenergic receptor and uncoupling protein-1 genes on weight loss and weight maintenance in Finnish women. *J. Clin. Endocrinol. Metab.* 83 4246–4250. 10.1210/jcem.83.12.5339 9851758

[B13] Forga L. Corbalan M. Marti A. Fuentes C. Martinez-Gonzalez M. A. Martinez A. (2003). [Influence of the polymorphism 03826 A – > G in the UCP1 gene on the components of metabolic syndrome]. *An. Sist. Sanit. Navar.* 26 231–236. 12951617 10.23938/ASSN.0449

[B14] Gagnon J. Lago F. Chagnon Y. C. Perusse L. Naslund I. Lissner L. (1998). DNA polymorphism in the uncoupling protein 1 (UCP1) gene has no effect on obesity related phenotypes in the swedish obese subjects cohorts. *Int. J. Obes. Relat. Metab. Disord.* 22 500–505. 10.1038/sj.ijo.0800613 9665669

[B15] Gbi research (2013). *Cardiovascular Disease Market: US to Lead Modest Growth, Forecasts GBI Research*. Available at: http://gbiresearch.com/media-center/press-releases/cardiovascular-disease-market-us-to-lead-modest-growth-forecasts-gbi-research [accessed 1 February, 2016]

[B16] Golozoubova V. Hohtola E. Matthias A. Jacobsson A. Cannon B. Nedergaard J. (2001). Only UCP1 can mediate adaptive nonshivering thermogenesis in the cold. *FASEB J.* 15 2048–2050. 10.1096/fj.00-0536fje 11511509

[B17] Grundy S. M. Cleeman J. I. Merz C. N. Brewer H. B. Jr. Clark L. T. Hunninghake D. B. (2004). Implications of recent clinical trials for the national cholesterol education program adult treatment panel III guidelines. *J. Am. Coll. Cardiol.* 44 720–732. 10.1016/j.jacc.2004.07.001 15358046

[B18] Gunawardana S. C. Piston D. W. (2012). Reversal of type 1 diabetes in mice by brown adipose tissue transplant. *Diabetes Metab. Res. Rev.* 61 674–682. 10.2337/db11-0510 PMC3282804 22315305

[B19] Hamann A. Tafel J. Busing B. Munzberg H. Hinney A. Mayer H. (1998). Analysis of the uncoupling protein-1 (UCP1) gene in obese and lean subjects: identification of four amino acid variants. *Int. J. Obes. Relat. Metab. Disord.* 22 939–941. 10.1038/sj.ijo.0800725 9756256

[B20] Hayakawa T. Nagai Y. Taniguchi M. Yamashita H. Takamura T. Abe T. (1999). Phenotypic characterization of the beta3-adrenergic receptor mutation and the uncoupling protein 1 polymorphism in Japanese men. *Metabolism* 48 636–640. 10.1016/S0026-0495(99)90063-X 10337866

[B21] Herrmann S. M. Wang J. G. Staessen J. A. Kertmen E. Schmidt-Petersen K. Zidek W. (2003). Uncoupling protein 1 and 3 polymorphisms are associated with waist-to-hip ratio. *J. Mol. Med. (Berl)* 81 327–332. 10.1007/s00109-003-0431-1 12756473

[B22] Holvoet P. (2012). Stress in obesity and associated metabolic and cardiovascular disorders. *Scientifica (Cairo)* 2012:205027 10.6064/2012/205027 PMC3820434 24278677

[B23] Huntington M. K. Shewmake R. A. (2010). Weight-loss supplements: what is the evidence? *S. D. Med.* 63 205–207. 20853590

[B24] Ioannidis J. P. Ntzani E. E. Trikalinos T. A. Contopoulos-Ioannidis D. G. (2001). Replication validity of genetic association studies. *Nat. Genet.* 29 306–309. 10.1038/ng749 11600885

[B25] Jia J. J. Tian Y. B. Cao Z. H. Tao L. L. Zhang X. Gao S. Z. (2010). The polymorphisms of UCP1 genes associated with fat metabolism, obesity and diabetes. *Mol. Biol. Rep.* 37 1513–1522. 10.1007/s11033-009-9550-2 19444646

[B26] Keipert S. Ost M. Chadt A. Voigt A. Ayala V. Portero-Otin M. (2013). Skeletal muscle uncoupling-induced longevity in mice is linked to increased substrate metabolism and induction of the endogenous antioxidant defense system. *Am. J. Physiol. Endocrinol. Metab.* 304 E495–E506. 10.1152/ajpendo.00518.2012 23277187

[B27] Kiec-Wilk B. Wybranska I. Malczewska-Malec M. Leszczynska-Golabek L. Partyka L. Niedbal S. (2002). Correlation of the -3826A > G polymorphism in the promoter of the uncoupling protein 1 gene with obesity and metabolic disorders in obese families from southern Poland. *J. Physiol. Pharmacol.* 53 477–490. 12375583

[B28] Kogure A. Yoshida T. Sakane N. Umekawa T. Takakura Y. Kondo M. (1998). Synergic effect of polymorphisms in uncoupling protein 1 and beta3-adrenergic receptor genes on weight loss in obese Japanese. *Diabetologia* 41:1399 10.1007/s001250051084 9833952

[B29] Malczewska-Malec M. Wybranska I. Leszczynska-Golabek I. Partyka L. Hartwich J. Jabrocka A. (2004). Analysis of candidate genes in Polish families with obesity. *Clin. Chem. Lab. Med.* 42 487–493. 10.1515/cclm.2004.083 15202783

[B30] Mathers C. D. Boerma T. Ma Fat D. (2009). Global and regional causes of death. *Br. Med. Bull.* 92 7–32. 10.1093/bmb/ldp028 19776034

[B31] Mattson M. P. (2010). Perspective: does brown fat protect against diseases of aging? *Ageing Res. Rev.* 9 69–76. 10.1016/j.arr.2009.11.004 19969105 PMC2818667

[B32] Mottagui-Tabar S. Hoffstedt J. Brookes A. J. Jiao H. Arner P. Dahlman I. (2008). Association of ADRB1 and UCP3 gene polymorphisms with insulin sensitivity but not obesity. *Horm. Res.* 69 31–36. 10.1159/000111793 18059082

[B33] Nagai N. Sakane N. Fujishita A. Fujiwara R. Kimura T. Kotani K. (2007). The -3826 A – > G variant of the uncoupling protein-1 gene diminishes thermogenesis during acute cold exposure in healthy children. *Obes. Res. Clin. Pract.* 1 I–II. 10.1016/j.orcp.2007.02.001 24351450

[B34] Nakano T. Shinka T. Sei M. Sato Y. Umeno M. Sakamoto K. (2006). A/G heterozygote of the A-3826G polymorphism in the UCP-1 gene has higher BMI than A/A and G/G homozygote in young Japanese males. *J. Med. Invest.* 53 218–222. 10.2152/jmi.53.218 16953057

[B35] Nichols M. Townsend N. Luengo-Fernandez R. Leal J. Gray A. Scarborough P. (2012). *European Cardiovascular Disease Statistics 2012.* Belgium: European Heart Network and European Society of Cardiology.

[B36] Nieters A. Becker N. Linseisen J. (2002). Polymorphisms in candidate obesity genes and their interaction with dietary intake of n-6 polyunsaturated fatty acids affect obesity risk in a sub-sample of the EPIC-Heidelberg cohort. *Eur. J. Nutr.* 41 210–221. 10.1007/s00394-002-0378-y 12395215

[B37] Oh H. H. Kim K. S. Choi S. M. Yang H. S. Yoon Y. (2004). The effects of uncoupling protein-1 genotype on lipoprotein cholesterol level in Korean obese subjects. *Metabolism* 53 1054–1059. 10.1016/j.metabol.2004.02.014 15281018

[B38] Ost M. Werner F. Dokas J. Klaus S. Voigt A. (2014). Activation of AMPKalpha2 is not crucial for mitochondrial uncoupling-induced metabolic effects but required to maintain skeletal muscle integrity. *PLoS ONE* 9:e94689 10.1371/journal.pone.0094689 PMC3986237 24732703

[B39] Poirier P. Giles T. D. Bray G. A. Hong Y. Stern J. S. Pi-Sunyer F. X. (2006). Obesity and cardiovascular disease: pathophysiology, evaluation, and effect of weight loss. *Arterioscler. Thromb. Vasc. Biol.* 26 968–976. 10.1161/01.ATV.0000216787.85457.f3 16627822

[B40] Ramis J. M. Gonzalez-Sanchez J. L. Proenza A. M. Martinez-Larrad M. T. Fernandez-Perez C. Palou A. (2004). The Arg64 allele of the beta 3-adrenoceptor gene but not the -3826G allele of the uncoupling protein 1 gene is associated with increased leptin levels in the Spanish population. *Metabolism* 53 1411–1416. 10.1016/j.metabol.2004.06.006 15536594

[B41] Rose G. Crocco P. D’Aquila P. Montesanto A. Bellizzi D. Passarino G. (2011). Two variants located in the upstream enhancer region of human UCP1 gene affect gene expression and are correlated with human longevity. *Exp. Gerontol.* 46 897–904. 10.1016/j.exger.2011.07.011 21827845

[B42] Saito M. Okamatsu-Ogura Y. Matsushita M. Watanabe K. Yoneshiro T. Nio-Kobayashi J. (2009). High incidence of metabolically active brown adipose tissue in healthy adult humans: effects of cold exposure and adiposity. *Diabetes Metab. Res. Rev.* 58 1526–1531. 10.2337/db09-0530 PMC2699872 19401428

[B43] Sherry S. T. Ward M. H. Kholodov M. Baker J. Phan L. Smigielski E. M. (2001). dbSNP: the NCBI database of genetic variation. *Nucleic Acids Res.* 29 308–311. 10.1093/nar/29.1.308 11125122 PMC29783

[B44] Sivenius K. Valve R. Lindi V. Niskanen L. Laakso M. Uusitupa M. (2000). Synergistic effect of polymorphisms in uncoupling protein 1 and beta3-adrenergic receptor genes on long-term body weight change in Finnish type 2 diabetic and non-diabetic control subjects. *Int. J. Obes. Relat. Metab. Disord.* 24 514–519. 10.1038/sj.ijo.0801194 10805511

[B45] Slavich G. M. Cole S. W. (2013). The emerging field of human social genomics. *Clin. Psychol. Sci.* 1 331–348. 10.1177/2167702613478594 23853742 PMC3707393

[B46] Sramkova D. Krejbichova S. Vcelak J. Vankova M. Samalikova P. Hill M. (2007). The UCP1 gene polymorphism A-3826G in relation to DM2 and body composition in Czech population. *Exp. Clin. Endocrinol. Diabetes* 115 303–307. 10.1055/s-2007-977732 17516293

[B47] Takahashi R. Imamura A. Yoshikane M. Suzuki M. Murakami R. Cheng X. W. (2010). Very small low-density lipoprotein cholesterol level is a determinant of arterial stiffness in men with impaired glucose metabolism. *J. Atheroscler. Thromb.* 17 1282–1289. 10.5551/jat.5272 20834193

[B48] Tsuboyama-Kasaoka N. Tsunoda N. Maruyama K. Takahashi M. Kim H. Ikemoto S. (1998). Up-regulation of uncoupling protein 3 (UCP3) mRNA by exercise training and down-regulation of UCP3 by denervation in skeletal muscles. *Biochem. Biophys. Res. Commun.* 247 498–503. 10.1006/bbrc.1998.8818 9642158

[B49] Urhammer S. A. Fridberg M. Sorensen T. I. Echwald S. M. Andersen T. Tybjaerg-Hansen A. (1997). Studies of genetic variability of the uncoupling protein 1 gene in Caucasian subjects with juvenile-onset obesity. *J. Clin. Endocrinol. Metab.* 82 4069–4074. 10.1210/jcem.82.12.4414 9398715

[B50] van Marken Lichtenbelt W. D. Schrauwen P. (2011). Implications of nonshivering thermogenesis for energy balance regulation in humans. *Am. J. Physiol. Regul. Integr. Comp. Physiol.* 301 R285–R296. 10.1152/ajpregu.00652.2010 21490370

[B51] van Marken Lichtenbelt W. D. Vanhommerig J. W. Smulders N. M. Drossaerts J. M. Kemerink G. J. Bouvy N. D. (2009). Cold-activated brown adipose tissue in healthy men. *N. Engl. J. Med.* 360 1500–1508. 10.1056/NEJMoa0808718 19357405

[B52] van Vliet-Ostaptchouk J. V. Nuotio M. L. Slagter S. N. Doiron D. Fischer K. Foco L. (2014). The prevalence of metabolic syndrome and metabolically healthy obesity in Europe: a collaborative analysis of ten large cohort studies. *BMC Endocr. Disord.* 14:9 10.1186/1472-6823-14-9 PMC3923238 24484869

[B53] Vimaleswaran K. S. Radha V. Deepa R. Mohan V. (2007). Absence of association of metabolic syndrome with PPARGC1A, PPARG and UCP1 gene polymorphisms in Asian Indians. *Metab. Syndr. Relat. Disord.* 5 153–162. 10.1089/met.2006.0032 18370824

[B54] Vissers D. Hens W. Taeymans J. Baeyens J. P. Poortmans J. Van Gaal L. (2013). The effect of exercise on visceral adipose tissue in overweight adults: a systematic review and meta-analysis. *PLoS ONE* 8:e56415 10.1371/journal.pone.0056415 PMC3568069 23409182

[B55] WHO (2007). *The Challenge of Obesity in the WHO European Region and the Strategies for Response.* Copenhagen: World Health Organization, Regional Office for Europe.

[B56] WHO (2011). *Global Atlas on Cardiovascular Disease Prevention and Control.* Geneva: World Health Organization;World Heart Federation; World Stroke Organization.

[B57] WHO (2014). *Global Status Report on Noncommunicable Diseases.* Geneva: World Health Organization.

[B58] WHO (2016a). *Cardiovascular Disease. World Health Organization.* Available at: http://www.who.int/cardiovascular_diseases/en/ [accessed 1 February, 2016]

[B59] WHO (2016b). *Diabetes. World Health Organization.* Available at: http://www.who.int/mediacentre/factsheets/fs312/en/ [accessed 2 August, 2016]

[B60] Wu J. Cohen P. Spiegelman B. M. (2013). Adaptive thermogenesis in adipocytes: is beige the new brown? *Genes Dev.* 27 234–250. 10.1101/gad.211649.112 23388824 PMC3576510

[B61] Yoshida T. Kato K. Fujimaki T. Yokoi K. Oguri M. Watanabe S. (2009). Association of genetic variants with chronic kidney disease in Japanese individuals. *Clin. J. Am. Soc. Nephrol.* 4 883–890. 10.2215/cjn.04350808 19406964 PMC2676181

